# Sex Differences of Microglia and Synapses in the Hippocampal Dentate Gyrus of Adult Mouse Offspring Exposed to Maternal Immune Activation

**DOI:** 10.3389/fncel.2020.558181

**Published:** 2020-10-15

**Authors:** Chin Wai Hui, Haley A. Vecchiarelli, Étienne Gervais, Xiao Luo, Félix Michaud, Lisa Scheefhals, Kanchan Bisht, Kaushik Sharma, Lisa Topolnik, Marie-Ève Tremblay

**Affiliations:** ^1^Axe neurosciences, Centre de Recherche, Centre Hospitalier Universitarie de Qu-Université Laval, Québec, QC, Canada; ^2^Division of Medical Sciences, University of Victoria, Victoria, BC, Canada; ^3^Master Neuroscience and Cognition, Faculty of Science, Utrecht University, Utrecht, Netherlands; ^4^Department of Biochemistry, Microbiology, and Bioinformatics, Faculty of Science and Engineering, Université Laval, Québec, QC, Canada; ^5^Department of Molecular Medicine, Faculty of Medicine, Université Laval, Québec, QC, Canada; ^6^Neurology and Neurosurgery Department, McGill University, Montréal, QC, Canada; ^7^Department of Biochemistry and Molecular Biology, The University of British Columbia, Vancouver, BC, Canada

**Keywords:** microglia, schizophrenia, maternal immune activation, complement, dentate gyrus, phagocytosis, mice

## Abstract

Schizophrenia is a psychiatric disorder affecting ∼1% of humans worldwide. It is earlier and more frequently diagnosed in men than woman, and men display more pronounced negative symptoms together with greater gray matter reductions. Our previous findings utilizing a maternal immune activation (mIA) mouse model of schizophrenia revealed exacerbated anxiety-like behavior and sensorimotor gating deficits in adult male offspring that were associated with increased microglial reactivity and inflammation in the hippocampal dentate gyrus (DG). However, both male and female adult offspring displayed stereotypy and impairment of sociability. We hypothesized that mIA may lead to sex-specific alterations in microglial pruning activity, resulting in abnormal synaptic connectivity in the DG. Using the same mIA model, we show in the current study sex-specific differences in microglia and synapses within the DG of adult offspring. Specifically, microglial levels of cluster of differentiation (CD)68 and CD11b were increased in mIA-exposed females. Sex-specific differences in excitatory and inhibitory synapse densities were also observed following mIA. Additionally, inhibitory synaptic tone was increased in DG granule cells of both males and females, while changes in excitatory synaptic transmission occurred only in females with mIA. These findings suggest that phagocytic and complement pathways may together contribute to a sexual dimorphism in synaptic pruning and neuronal dysfunction in mIA, and may propose sex-specific therapeutic targets to prevent schizophrenia-like behaviors.

## Introduction

Schizophrenia is a psychiatric disorder that affects ∼1% of humankind. It is more frequently diagnosed in men than women; additionally, men are more likely to have an earlier age of disease onset and experience more negative symptoms (Mendrek and Mancini-Marïe, 2016). Microglia, yolk-sac derived innate immune cells that inhabit the brain early during development, potentially contribute to these sex differences. Microglia are required for proper brain development, plasticity and homeostasis (Tian et al., 2017). Their pruning of synapses can be mediated through the complement (C) 3/C1q pathway (Stevens et al., 2007; Paolicelli et al., 2011; Schafer et al., 2012), which may contribute to an inadequate synaptic maintenance when abnormally active in disease (Druart and Le Magueresse, 2019). In response to changes in homeostasis, microglia can acquire reactive phenotypes, in which they increase their secretion of inflammatory cytokines and neurotrophic factors, as well as modify their phagocytic activity (Kettenmann et al., 2011; Cherry et al., 2014). Microglial alterations were reported throughout the *post-mortem* brains of schizophrenia patients, specifically an increase in ionized calcium binding adaptor molecule (IBA) 1-immunopositive (+) cells (Mongan et al., 2019). Animal models of schizophrenia induced by maternal immune activation (mIA) show microglial alterations (Juckel et al., 2011; Ratnayake et al., 2012; Pratt et al., 2013; Van den Eynde et al., 2014; Giovanoli et al., 2016). Our previous work revealed overall sex differences in microglial density, and, utilizing a polyinosinic:polycytidylic acid (poly I:C) mIA mouse model, increased morphological index (soma area/arborization area) and interactions with synapses in the hippocampal dentate gyrus (DG) of adult male offspring, correlating with schizophrenia-like behavioral deficits (Hui et al., 2018).

In addition to sex differences in schizophrenia, there is significant sexual dimorphism in embryonic migration, gene expression and function of microglia throughout life (Bordeleau et al., 2019). Microglial density and morphology differ between sexes in the rat hippocampus: at embryonic day (E) 17 there are no sex differences, but at postnatal (P) 0, females show increased numbers of ameboid and stout microglia; whereas at P4, males have more ameboid, stout and non-ramified microglia; then at P30 and P60, females display more abundant microglia with longer or ramified processes (Schwarz et al., 2012). Microglial phagolysosomal activity is sexually dimorphic in the mouse hippocampus: females at P3 have greater numbers of microglia with phagocytic cups in which cluster of differentiation (CD) 68, a marker of phagolysosomal activity, colocalizes with neuronal elements (Nelson et al., 2017); while females at P8 and males at P28 have greater staining intensity for CD68 (Weinhard et al., 2018). Based on these findings, we hypothesized that mIA induced with poly I:C may lead to sex-specific alterations in microglial phagocytosis, resulting in abnormal synaptic pruning in the DG, leading to the behavioral outcomes observed at adulthood. The DG is a region that is greatly affected by schizophrenia, as neuroimaging reports show reduced volumes in patients and animal models (Tavitian et al., 2019). There is also a potential arrestment of maturation of granule cells (GCs) in the DG in both patients with and potentially in animal models of schizophrenia (Tavitian et al., 2019), which may be the result of defective synaptic elimination (Feinberg, 1982). We examined in this study changes in microglial phagocytic markers, complement system, neuronal oxidative stress, as well as synaptic density and activity in the DG of adult male and female mouse offspring exposed to mIA at E9.5.

## Materials and Methods

### Animals

Animals were housed under a 12 h light–dark cycle at 22–25°C, with free access to food and water. Experimental animals were generated from C57Bl/6 mice (Charles River). All experiments were approved by Université Laval’s animal ethics committees, according to the Canadian Council on Animal Care’s guidelines.

### Poly I:C Maternal Immune Activation Model

Infection was simulated by intraperitoneal (i.p) injection of poly I:C potassium salt (Sigma-Aldrich, P9582) (5 mg/kg in sterile saline) at E9.5 as described (Hui et al., 2018). A vehicle control group received sterile saline. Each analysis included animals from three to five litters. Two to five mice per sex/group were housed together until behavioral testing from P60 to P80 followed by sacrifice 48 h later (cohorts 1 and 2; detailed in Hui et al., 2018), or sacrifice at P80–90 (cohort 3 for electrophysiology). Cohorts 1 and 2 mice were anesthetized with ketamine-xylazine (80/10 mg/mL), then cohort 1 mice were transcardially perfused with ice-cold phosphate buffered saline (PBS; 50 mM, pH 7.4). Right hemisphere hippocampi were dissected and kept at −80°C for molecular studies. Left hemispheres were fixed by immersion in 4% paraformaldehyde (PFA; Electron Microscopy Sciences) at 4°C overnight. Cohort 2 mice were transcardially perfused with 0.2% glutaraldehyde (Electron Microscopy Sciences)/4% PFA.

### Fluorescent Immunohistochemistry

Left hemispheres were sectioned longitudinally at 30 μm using a cryostat. Sections containing the dorsal hippocampal DG (Bregma 0.36–1.00) (Paxinos and Watson, 1998) were processed for fluorescent immunohistochemistry. Sections were incubated in 0.1 M citrate buffer at 85°C for 8–10 min for antigen retrieval. After the slides cooled, they were washed with PBS and incubated in blocking buffer for 1 h at room temperature (RT). Sections were then incubated with primary antibodies overnight at 4°C, rinsed in PBS and incubated with secondary antibodies for 2 h at RT (see Supplementary Table S1 for antibody information). Sections were then washed in PBS, counter-stained with 4′,6-diamidino-2-phenylindole (DAPI) (1:20,000; Thermo Fisher Scientific) and coverslipped in Fluoromount-G (SouthernBiotech).

### Confocal Imaging

All imaging analyses were performed by an investigator unaware of experimental conditions.

#### Confocal Imaging and Quantitative Analysis of Microglia, Astrocytes and Neurons

Using a Quorum WaveFX Spinning disc confocal microscope, *Z*-stacks (∼50 slices, Δz = 0.5 μm) were acquired at 20x magnification with an ORCA-R2 camera (Hamamatsu, 1,344 × 1,024 pixels) in two regions of interest (ROI) in the DG for each section, covering the GCs layer (GL) and polymorphic layer (PL). Two sections per animal were imaged and the results averaged. Focus stacking was performed with Volocity (Version 5.4, PerkinElmer). Colocalization of CD68 in IBA1+ microglia was assayed with ImageJ (National Institutes of Health). Twenty to thirty microglia were analyzed per animal. The analysis focused on cell bodies, as staining was diffuse in processes. Data are presented as CD68 counts per IBA1+ microglial cell. Signal intensity was assayed for glial fibrillary acidic protein (GFAP), C1q, C3, CD11b, and 3-nitrotyrosine (3-NT) using ImageJ. For each combination of antibodies, sections were imaged with the same laser intensity and were quantified using the area measurement tool keeping the threshold values constant. Data are expressed as percent area.

#### Confocal Imaging for Synaptic Density and Staining Intensity Analysis

Confocal imaging of the DG was performed with a Carl Zeiss LSM-800 laser scanning confocal microscope. *Z*-stacks (15 steps (Δz = 0.33 μm) were acquired using a Plan-Apochromat 63×/1.4 oil immersion objective (1,024 × 1,024 pixels). Two regions of interest (ROI) were imaged in the PL of two to three sections per animal. Synaptic puncta analysis was performed manually with ImageJ in locations devoid of DAPI staining. For inhibitory puncta analysis, a ROI of 30 × 30 μm was selected. Vesicular gamma aminobutyric acid (GABA) transporter (VGAT) and gephyrin puncta were counted individually before merging channels to count overlapping puncta. For excitatory puncta analysis, a ROI of 20 × 20 μm was selected. Maximum intensity projections were created from 4 *z*-steps. All puncta were counted in individual channels for Homer1 and vesicular glutamate transporter (VGLUT) 1 before merging channels to identify overlap. See Supplementary Materials and Methods for additional information.

### Tissue Preparation and Immunoperoxidase Signaling for Electron Microscopy

Fifty micrometer transverse sections from Bregma 2.12–1.64 (Paxinos and Watson, 1998) were cut using a vibratome. They were processed as described (Bisht et al., 2016). Briefly, sections were washed in Tris-buffered saline (TBS), quenched, and processed for CD68 immunostaining. They were blocked and incubated overnight in primary antibody (Supplementary Table S1) at 4°C, washed, incubated with secondary antibody for 1.5 h at RT, and then incubated with ABC Vectastain (1:100; Vector Laboratories, #PK−6100), diaminobenzidine (0.05%) and hydrogen peroxide (0.015%). The sections were post-fixed in 1% osmium tetroxide, dehydrated in ethanol, and embedded with Durcupan at 55°C for 72 h. Areas of the DG (PL and GL) were cut at 70–80 nm using an ultramicrotome (Leica Ultracut UC7). Ultrathin sections were examined at 80 kV with a FEI Tecnai Spirit G2 transmission electron microscope (TEM). Imaging was performed at 6,800X, using an ORCA-HR digital camera (10 MP; Hamamatsu). Dark neuronal cell bodies and dendrites were identified ultrastructurally as described previously (Tremblay et al., 2012; Audoy-Rémus et al., 2015). Their density was expressed as numbers per mm^2^ of screened tissue surface, using the systematic approach developed for dark microglia analysis (Bisht et al., 2016; Hui et al., 2018).

### Gene Expression Analysis Using Quantitative Real-Time PCR

Tissue was homogenized using the QIAzol lysis reagent (Qiagen) and total RNA was extracted by phenol-chloroform method. One microgram of total RNA was reverse transcribed into cDNA using the iScript cDNA synthesis kit (BioRad). Real-time PCR was performed with the SsoAdvanced universal SYBR Green supermix kit (BioRad) in a Lightcycler 480II (Roche). Primers (Supplementary Table S2) were designed using NCBI Primer Blast (Hui et al., 2018). Relative expression was calculated with the 2^–ΔΔCT^ method using *Gapdh* for normalization (Yuan et al., 2006).

### Electrophysiological Patch-Clamp Recordings

Cohort 3 mice were anesthetized with ketamine-xylazine (10/100 mg/mL) and perfused intracardially with an ice-cold sucrose-based solution (Supplementary Table S3). Transverse hippocampal slices (300 μm) were obtained using a vibratome (Microm HM 650V, Thermo Scientific) in the same sucrose-based solution oxygenated with 95%O_2_/5%CO_2_, and transferred into the heated oxygenated recovery solution (Supplementary Table S3) until use.

For electrophysiological recordings, slices were transferred to the recording chamber perfused continuously with an oxygenated artificial cerebrospinal fluid (ACSF) and maintained at near physiological temperature (Supplementary Table S3). The GCs within the DG were visually identified using a Nikon (Eclipse FN1) microscope with infrared differential interference contrast microscopy using a 40× (NA 0.8) water-immersion objective. Patch pipettes (3.5–6 MΩ) were made from borosilicate glass capillaries using a Flaming/Brown micropipette puller (Sutter Instrument Co.). Whole-cell patch-clamp recordings (5–10 min) were performed in voltage-clamp at –70 mV for miniature excitatory post-synaptic currents (mEPSCs) and at +10 mV for miniature inhibitory post-synaptic currents (mIPSCs). The passive membrane properties were taken immediately upon membrane rupture. The same intracellular solution (Supplementary Table S3) and ACSF with tetrodotoxin (TTX; 1 μM; Alomone Labs; for blocking action potentials) were utilized in all experiments. Data acquisition (digitized at 10 kHz; Digidata 1550A, Molecular Devices) was performed using the MultiClamp 700B amplifier and the pCLAMP 10.5 software (Molecular Devices). The resting membrane potential was taken immediately upon membrane rupture. The input resistance and the membrane capacitance were detected automatically during the first minute of whole-cell configuration using a 5 mV square pulse in Membrane Test of Clampex 10.7 software. More than 200 events per neuron were selected using an automated template search algorithm in Clampfit. All events were counted for frequency analysis as described previously (David and Topolnik, 2017). Anatomical analysis of biocytin-filled neurons was also performed (Supplementary Materials and Methods).

### Statistics

Data were analyzed using Prism (GraphPad, Version 8), Clampfit 10.7, Excel, IGOR Pro (Wavematrics) and Statistica (Tibco). Two-way ANOVAs with *post hoc* analyses using Bonferroni corrections were used to determine interactions between poly I:C exposure and sex effects. Outliers were determined using the ROUT test (Q = 0.1%; GraphPad) for cohort 1 and 2 animals. For cohort 3 animals, data distributions were tested for normality using Shapiro-Wilcoxon test, and Kolmogorov-Smirnov test was used for comparison of pooled data from individual cells. *p* < 0.05 was considered statistically significant. All data are reported as mean ± standard error of the mean (SEM).

## Results

### mIA Increases CD68 Levels in IBA1+ Microglia of Female but Not Male Offspring

**FIGURE 1:**
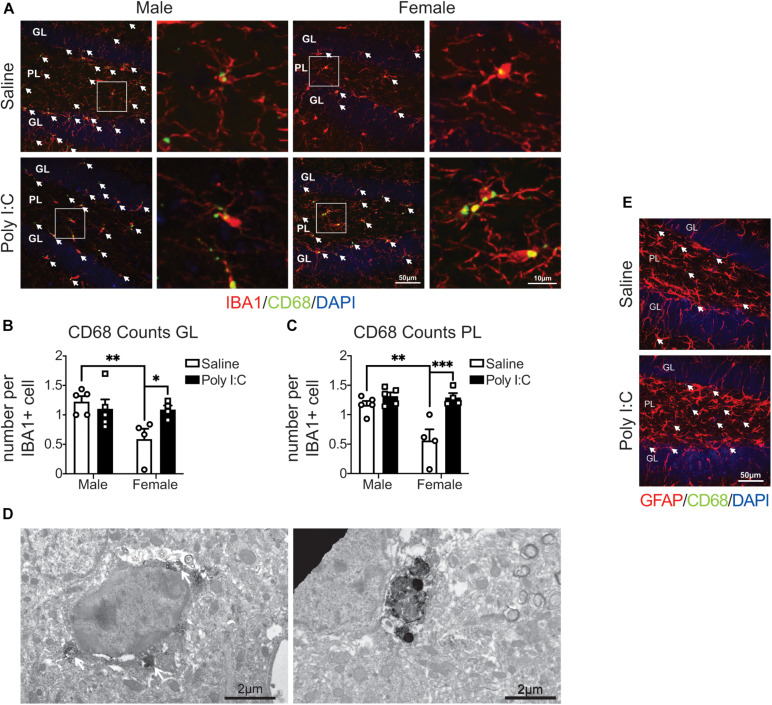
Male (left); female (right); saline (black outlined white bars with black outlined circles); poly I:C (black bars with black outline squares). *n* = 4–5 animals/group. ^∗^*p* < 0.05; ^∗∗^*p* < 0.01; ^∗∗∗^*p* < 0.001. CD68 levels were increased in IBA1+ microglia in the hippocampal DG of female mice exposed to maternal poly I:C. **(A)** Representative images (20x) (left of each pair) and 5x magnified pictures (100x) (right of each pair) of IBA1+ microglia containing CD68 puncta from each condition (left half: males, right half: females; saline: top, poly I:C: bottom). Each image was from the DG and includes the granule cell layer (GL) and polymorphic layer (PL). IBA1+ microglia are shown in red, CD68 puncta in green and DAPI stained nuclei in blue. Scale bar = 50 μm (20x) and 10 μm (100x). **(B)** CD68 puncta counts per IBA1+ microglia in the GL. There was an increase in CD68 counts in female offspring exposed to poly I:C [interaction: *F*(1, 14) = 5.41, *p* = 0.04; *p* = 0.05], but not male offspring (*p* > 0.99). There were less microglial CD68 puncta in females than males from saline exposed dams (*p* = 0.009), but not poly I:C exposed dams (*p* > 0.99). There was also a main effect of sex [*F*(1, 14) = 5.88, *p* = 0.03] with males having greater CD68 counts in total; but not between saline and poly I:C [*F*(1, 14) = 2.02, *p* = 0.18]. **(C)** CD68 counts per IBA1+ microglia in the PL. As with the GL, there was an increase in CD68 levels in females prenatally exposed to poly I:C [interaction: *F*(1, 14) = 7.99, *p* = 0.01; *p* = 0.0006], contrary to males (*p* > 0.99). Males whose dams were exposed to saline had greater CD68 levels than females (*p* = 0.002), but there were no sex differences in the poly I:C exposed offspring (*p* > 0.99). There was a main effect of sex [*F*(1, 14) = 9.14, *p* = 0.009], with males having greater CD68 counts overall, and of poly I:C administration also leading to greater CD68 puncta counts overall [*F*(1, 14) = 18.09, *p* = 0.0008]. **(D)** Representative electron micrograph (left) confirming the association of CD68 to lysosomes inside microglial cell bodies in mice exposed to mIA. White arrows indicate CD68 signal surrounding lysosomes and white asterisks represent CD68 signal adjacent to extracellular digestion. Scale bar = 2 μm. Representative electron micrograph (right) (6,800x) showing CD68 staining in a microglial process. Scale bar = 2 μm. **(E)** CD68 signal did not colocalize with GFAP+ astrocytes. Representative image from saline and poly I:C conditions. Each image was taken of the DG and included the GL and PL. GFAP+ astrocytes are show in red, CD68 puncta in green and indicated by white arrows, and DAPI stained nuclei in blue. Scale bar = 50 μm.

We first assessed microglial phagolysosomal activity by immunofluorescent confocal microscopy against CD68 in the DG of adult offspring. In response to mIA, IBA1+ microglia had increased numbers of CD68 puncta in female vs. male offspring, in both the GL and PL (Figures 1A–C), indicating an increased phagocytic activity in the DG of female offspring. The baseline levels of CD68 also differed between sexes, with males showing greater immunoreactivity than females in the GL and PL of saline offspring (Figures 1A–C). Immunocytochemical TEM confirmed the localization of CD68 protein in microglial soma and processes from mIA and control offspring, across the GL and PL. CD68 staining was particularly found on endosomes, lysosomes and the plasma membrane, suggesting extracellular digestion or “exophagy” (Figure 1D). CD68 was not found, however, in GFAP+ astrocytes (Figure 1E). These data indicate that microglia from mIA-exposed female but not male offspring display increased phagolysosomal activity in the DG, with more pronounced changes observed in the PL.

### mIA Is Associated With Changes in the Complement System in Male and Female Offspring

**FIGURE 2:**
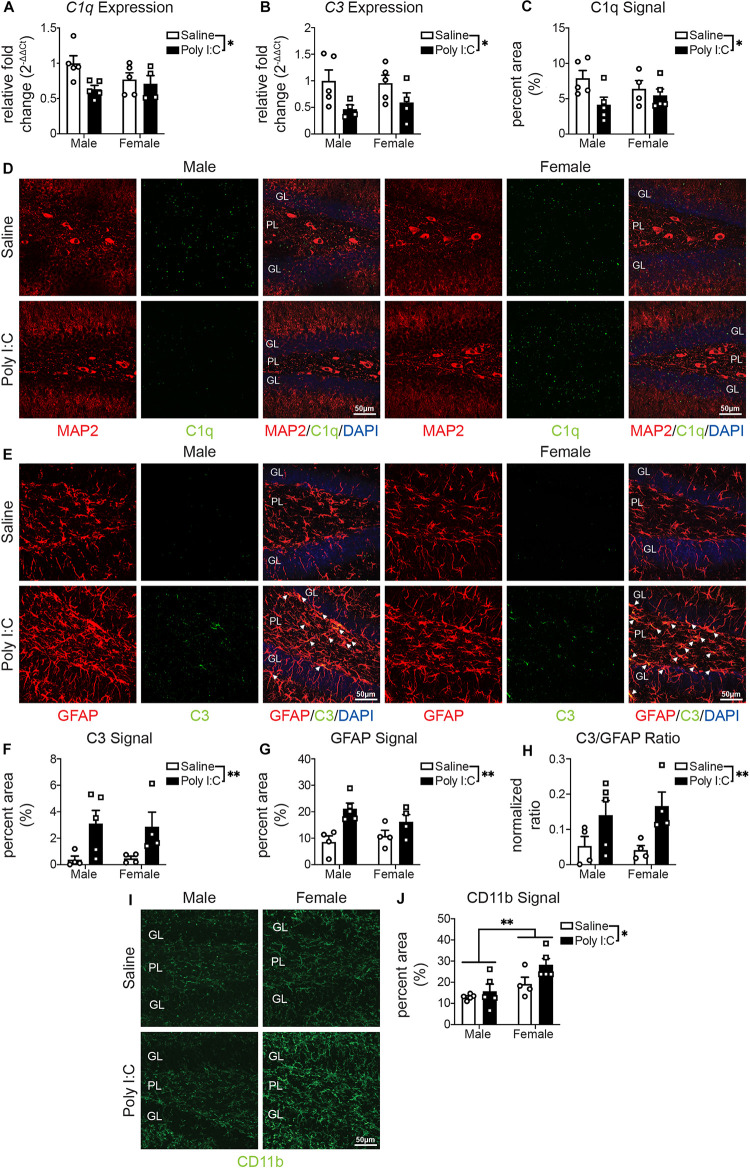
Male (left); female (right); saline (black outlined white bars with black outlined circles); poly I:C (black bars with black outline squares). *n* = 4–5 animals per group. *p < 0.05, **p < 0.01. mIA led to decreased expression of *C1q* and *C3* in the hippocampus, reduced levels of C1q and increased levels of C3 in the DG in a sex independent manner, while increasing CD11b levels in the DG of female offspring. **(A)** Relative expression of *C1q* in the DG. There was an overall reduction from maternal poly I:C exposure [*F*(1, 15) = 5.19, *p* = 0.04]. There was not an overall effect of sex [*F*(1, 15) = 0.65, *p* = 0.43] or interaction between the two factors [*F*(1, 15) = 2.70, *p* = 0.12]. **(B)** Relative expression of *C3* in the DG. As with C1q, there was an overall reduction in offspring whose dams were exposed to poly I:C [*F*(1, 14) = 7.16, *p* = 0.02]. There was not an effect of sex [*F*(1, 14) = 0.06, *p* = 0.81] or an interaction between the two [*F*(1, 14) = 0.25, *p* = 0.63]. **(C)** C1q levels were lower in offspring whose dams were exposed to poly I:C [*F*(1, 15) = 4.89, *p* = 0.04]. There was not an overall effect of sex [*F*(1, 15) = 0.009, *p* = 0.93] or interaction between the two [*F*(1, 15) = 1.81, *p* = 0.20]. **(D)** Representative images (left half: males, right half: females; saline: top, poly I:C: bottom) of C1q levels in the hippocampal DG (MAP2 (neurons; red, left); C1q (green, middle); merged [MAP2 (red)/C1q (green)/DAPI stained nuclei (blue); right)]. Each image was from the DG and includes the granule cell layer (GL) and polymorphic layer (PL). Scale bar = 50 μm. **(E)** Representative images (left half: males, right half: females; saline: top, poly I:C: bottom) of C3 levels in the DG [GFAP (astrocytes; red, left); C3 (green, middle); merged (GFAP (red)/C3 (green)/DAPI stained nuclei (blue); right)]. Each image was from the DG and includes the GL and PL. Scale bar = 50 μm. **(F)** C3 levels were greater in offspring from poly I:C exposed mothers [*F*(1, 13) = 10.01, *p* = 0.008]. There was not an effect of sex [*F*(1, 13) = 0.008, *p* = 0.93] or an interaction between the two [*F*(1, 13) = 0.04, *p* = 0.84]. **(G)** GFAP levels were also greater in offspring following maternal poly I:C administration [*F*(1, 13) = 14.65, *p* = 0.002]. There was not an effect of sex [*F*(1, 13) = 2.37, *p* = 0.15] or interaction between the two [*F*(1, 13) = 0.33, *p* = 0.57]. **(F)** The levels of C3 in GFAP+ positive cells was also greater in offspring when dams were exposed to poly I:C [*F*(1, 13) = 9.65, *p* = 0.008]. There was not an effect of sex [*F*(1, 13) = 0.31, *p* = 0.59] or an interaction between the two [*F*(1, 13) = 0.04, *p* = 0.84]. **(H)** Representative images of CD11b (green) levels in the DG (left: males, right half: females; saline: top, poly I:C: bottom). Each image was from the DG and includes the GL and PL. Scale bar = 50 μm. **(I)** CD11b levels were greater in offspring following maternal poly I:C administration [*F*(1, 15) = 4.61, *p* = 0.05]; furthermore, female offspring, from dams who were exposed to either saline or poly I:C had greater CD11b levels [*F*(1, 15) = 11.57, *p* = 0.004]. There was not an interaction between the two [*F*(1, 15) = 1.34, *p* = 0.26].

One hemisphere of the whole hippocampus of adult offspring was examined by quantitative real-time PCR. Overall, there were no changes with mIA or between sexes in the expression of genes related to synaptic development or maintenance (*Stx1a*, *Vamp2*, *Snap25*, *Bdnf*, *Fmr1*, and *Shank2*) (Supplementary Table S4). For genes involved in phagocytosis or synaptic pruning, there was a reduction with mIA in the expression of complement system components *C1q* (Figure 2A) and *C3* (Figure 2B), without changes in the expression of *Axl*, *MerTK*, *Gas6*, and *C4a* (Supplementary Table S4).

We then used immunofluorescent staining to examine by confocal microscopy protein levels of complement components in the DG of adult offspring. C1q staining did not localize to IBA1+ microglia (Supplementary Figure S1A) or GFAP+ astrocytes (Supplementary Figure S1B). Complementary to gene expression, C1q protein levels in microtubule-associated protein (MAP) 2+ neurons were lower in mIA-exposed offspring (Figures 2C–D). C3 protein levels, unlike gene expression data, were also greater in mIA-exposed offspring (Figures 2E–H). Confocal microscopy additionally revealed that C3 protein localizes to GFAP+ astrocytes (Figure 2E), while GFAP staining intensity was increased in mIA-exposed offspring (Figure 2G). Proteins levels of complement receptor 3 (CR3)’s component CD11b were assayed, revealing increased staining in mIA-exposed offspring and overall in females (Figures 2I,J). Together these data reveal alterations of the complement system following mIA, without a clear pattern emerging regarding sex differences, except for greater levels of CD11b in females.

### mIA Increases the Density of Excitatory and Inhibitory Synapses in Males While Reducing Excitatory Synapses in Females

**FIGURE 3:**
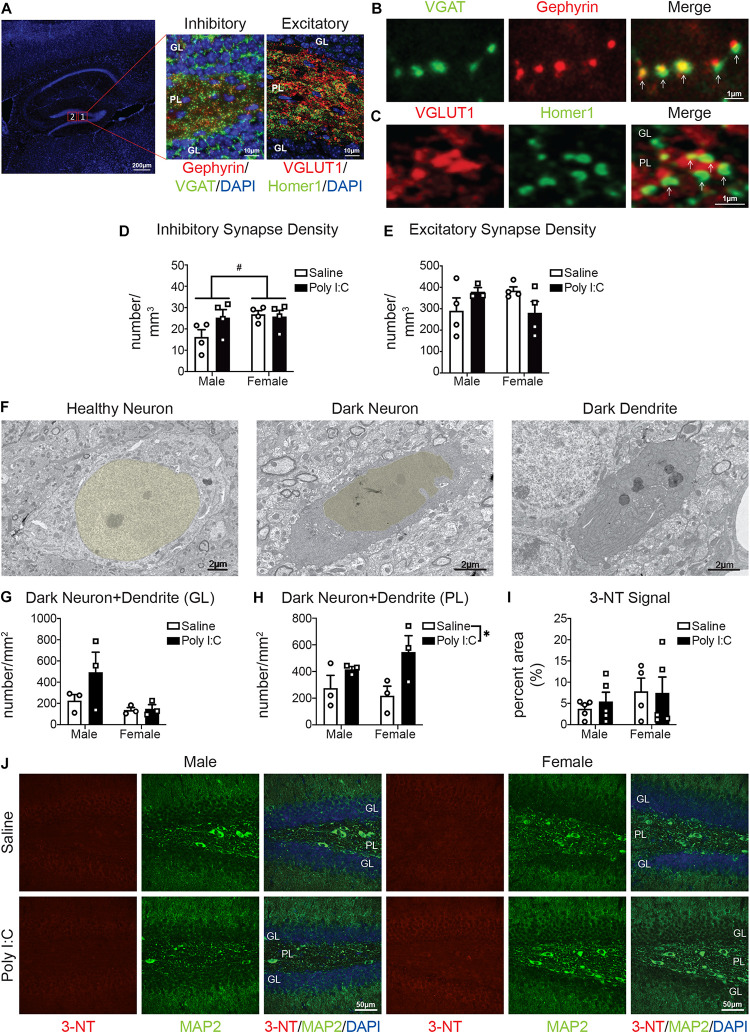
Male (left); female (right); saline (black outlined white bars with black outlined circles); poly I:C (black bars with black outline squares). *n* = 3–5 animals per group. ^#^*p* < 0.1; *p < 0.05. Maternal poly I:C administration led to increased excitatory and inhibitory synapse levels in the PL in males, but reduced excitatory synapse levels in females, as well as increased dark neurons in the PL, without changing levels of the neuronal oxidative stress marker, 3-NT in the DG. **(A)** Representative image of inhibitory and excitatory markers in the DG. Overview of the DG (left) (DAPI stained nuclei in blue; scale bar = 200 μm). Magnified images (63x) containing the polymorphic layer (PL) of the DG. The middle shows a representative image of inhibitory puncta (VGAT in green; Gephyrin in red; and DAPI stained nuclei in blue; scale bar = 10 μm). The right image shows a representative image of excitatory puncta (VGLUT in red; Homer1 in green; DAPI stained nuclei in blue; scale bar = 10 μm). **(B)** Representative images (63x) of components of inhibitory synapses (left, VGAT in green; middle, Gephyrin in red; right, merged; scale bar = 1 μm). **(C)** Representative images (63x) of components of excitatory synapses (left, VGLUT in red; middle, Homer1 in green; right, merged; scale bar = 1 μm). **(D)** There were potential baseline sex differences in inhibitory synapses between males and females [main effect, sex, *F*(1, 12) = 3.31, *p* = 0.09; *p* = 0.06], with males having less than females, but not following mIA (*p* > 0.99). There was no significant effect of poly I:C on excitatory synapses [interaction, *F*(1, 12) = 2.72, *p* = 0.13]; main effect, poly I:C [*F*(1, 12) = 1.71, *p* = 0.22]; males (*p* = 0.12); females (*p* > 0.99). **(E)** There were potential differences in excitatory synapses between males and females and following maternal poly I:C administration [interaction, *F*(1, 12) = 4.61, *p* = 0.06], although specific comparisons revealed no significant changes (males saline vs. poly I:C, *p* = 0.41; females saline vs. poly I:C, *p* = 0.23; saline males vs. females, *p* = 0.30; poly I:C males vs. females, *p* = 0.32), the pattern was males had lower levels compared to females at baseline, which was increased following poly I:C, whereas females had reduced levels following poly I:C. **(F)** Representative electron micrographs (6,800x) from the PL showing a healthy neuron (nucleus pseudocolored yellow; left), dark neuron (nucleus pseudocolored yellow; middle) and a dark dendrite (right). Scale bar = 2 μm. **(G)** There was no overall effect of maternal poly I:C administration or sex differences on the density of dark neuronal soma/dendrites in the granule cell layer (GL) of the DG [interaction, *F*(1, 8) = 1.57, *p* = 0.25; main effect of sex, *F*(1, 8) = 4.53, *p* = 0.07; main effect of poly I:C, *F*(1, 8) = 1.88, *p* = 0.21]. **(H)** In the polymorphic layer (PL) of the DG, maternal poly I:C administration increased dark neuronal soma and dendrites numbers [main effect, *F*(1, 8) = 7.47, *p* = 0.03]. There was not an effect of sex [main effect, *F*(1, 8) = 0.18, *p* = 0.68] or interaction between the two [*F*(1, 8) = 1.19, *p* = 0.31]. **(I)** There was no overall effect of maternal poly I:C administration or sex differences on the levels of 3-NT in the DG [interaction, *F*(1, 15) = 0.15, *p* = 0.70; main effect of sex, *F*(1, 15) = 1.31, *p* = 0.27; main effect of poly I:C, *F*(1, 15) = 0.07, *p* = 0.80]. **(J)** Representative images from each condition (left half: males, right half: females; saline: top, poly I:C: bottom) of dentate gyri with immunohistochemistry for 3-NT (oxidative stress marker; red, left), MAP2 (neuronal markers; green, middle) and merged [3-NT (red)/MAP2 (green)/DAPI (nuclei; blue)]. Each image was from the DG and includes the GL and PL. Scale bar = 50 μm.

To provide functional insights into these changes in phagolysosomal activity and the complement system, we measured synaptic density by immunofluorescent confocal microscopy, in the PL of adult offspring. Inhibitory synapses were assessed via colocalization of VGAT (presynaptic) and gephyrin (post-synaptic) (Figures 3A,B). Excitatory synapses were assessed by colocalization of VGLUT1 (presynaptic) and Homer1 (post-synaptic) (Figures 3A,C). There was potential increased density (Figure 3D) and staining intensity (Supplementary Figures S3A,B) of inhibitory synapses with mIA in males but not females; additionally, there were potential baseline sex differences in inhibitory synapses, with males having fewer than females (Figure 3D). For excitatory synapses, between males and females and following mIA, there was a potential interaction; pattern-wise, males showed an increase, and females a decrease in their density (Figure 3E); for staining intensity, there was only an effect of mIA on VGLUT1 but no effect on Homer1 (Supplementary Figures S2C,D). Similarly, there were baseline sex differences, with females having more excitatory synapses than males (Figure 3E). These data indicate that following mIA, there is a dynamic synaptic reorganization taking place in the PL, where inhibitory and excitatory synapses both increase in male offspring, and where inhibitory synapses are unchanged while excitatory synapses decrease in female offspring.

### mIA Increases Dark Neurons Without Affecting 3-NT Levels in Both Sexes

To determine whether these changes of synapses are associated with altered neuronal health, we next examined the occurrence of dark neurons in the DG of adult offspring. Dark neurons are recognized by cellular stress markers: increased nuclear and cytoplasmic condensation, loss of heterochromatin pattern, as well as dilation of endoplasmic reticulum/Golgi apparatus; and are not necrotic or apoptotic (Turmaine et al., 2000; Tremblay et al., 2012; Henry et al., 2018). In the GL, the density of dark neuronal soma/dendrites was unchanged following mIA (Figures 3F,G). In the PL, however, there was a general effect of mIA increasing the numbers of dark neuronal soma/dendrites (Figures 3F,H). By immunofluorescent confocal microscopy, the levels of 3-NT, a biomarker of reactive nitrogen species formation (Walker et al., 2001), was also examined in the DG of adult offspring. 3-NT was only observed in MAP2+ neurons. There was no effect of mIA on 3-NT levels in both males and females (Figures 3I,J). These data combined point to potential neuronal stress following mIA, without any change in the oxidative stress marker 3-NT in neurons of the DG.

### mIA Induces Sex-Specific Alterations in mIPSCs and mEPSCs of GCs

**FIGURE 4:**
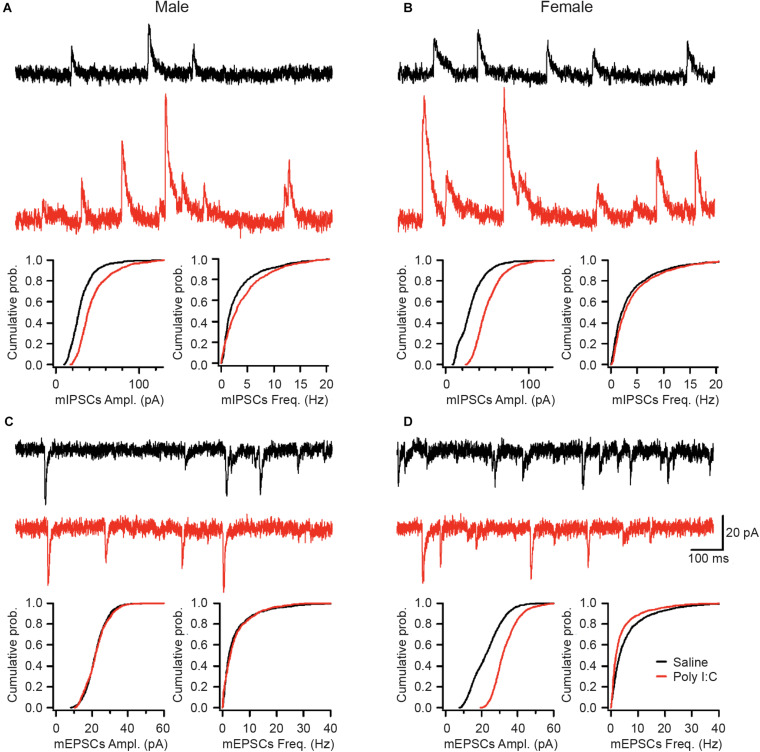
Saline (top, black; male: *n* = 7 cells/5 mice; female: *n* = 9 cells/6 mice) or poly I:C (bottom, red; male: *n* = 5 cells/4 mice; female: *n* = 10 cells/5 mice). mIA led to increased mIPSC amplitude in GC of both males and females, together with increased mIPSC frequency in males, while increasing mEPSC amplitude but decreasing mEPSC frequency in females, without altering mEPSCs in males. **(A–D)** Representative traces from the GCs of the DG for miniature inhibitory post-synaptic currents (mIPSCs) in male **(A)** and female **(B)** offspring whose dams were exposed to saline or poly I:C. Representative traces from the DG GCs for miniature excitatory post-synaptic currents (mEPSCs) in male **(C)** and female **(D)** offspring whose dams were exposed to saline (top, black) or poly I:C (bottom, red). Scale bars indicate 20 pA (vertical) and 100 ms (horizontal). **(A)** Maternal poly I:C exposure increased the amplitude (median: saline, 27.6 pA; poly I:C, 38.6 pA; *p* < 0.0001) and frequency (median: saline, 1.75 Hz; poly I:C, 2.85 Hz; *p* < 0.0001) of mIPSCs in male offspring. **(B)** As with males, in female offspring of dams exposed to poly I:C, there was an increase in the amplitude (median: saline, 28.3 pA; poly I:C, 47.0 pA; *p* 0.0001), but not the frequency of mIPSCs (median: saline, 2.10 Hz; poly I:C, 2.60 Hz; *p* > 0.05). **(C)** There was no effect of maternal poly I:C administration in male offspring on either the amplitude (median: saline, 23.6 pA; poly I:C, 21.0 pA; *p* > 0.05) or frequency (median: saline, 5.4 Hz; poly I:C, 5.9 Hz; *p* > 0.05) of miniature excitatory post-synaptic currents (mEPSCs). **(D)** Maternal poly I:C exposure increased the amplitude (median: saline, 22.2 pA; poly I:C, 31.7 pA; *p* 0.0001) but decreased the frequency (median: saline, 3.25 Hz; poly I:C, 1.90 Hz; *p* 0.01) of mEPSCs in females.

Lastly, we investigated whether the observed changes in microglia and synapses are associated with functional or morphological alterations in the DG GCs of adult offspring. While no changes were revealed in the passive membrane properties (Supplementary Table S5) or the morphological features of GCs (Supplementary Figure S3), both mEPSCs and mIPSCs showed sex-specific differences. Specifically, while the amplitude of mIPSCs was increased in both males and females exposed to mIA, the frequency of mIPSCs was only increased in males (Figures 4A,B), consistent with a higher inhibitory synapse density in the PL of males (Figure 3A). Contrastingly, there was no effect of mIA on mEPSCs in males (Figure 4C). However, with mIA, mEPSC amplitude was increased while frequency was decreased in females (Figure 4D), which could be associated with a loss of excitatory synapses, and compensatory increase in conductance at the surviving contacts. Together, these results revealed an increased inhibitory tone in both sexes, as well as female offspring-specific changes in GCs excitatory synaptic transmission following maternal exposure to poly I:C.

## Discussion

This study highlights sex differences in microglial phagolysosomal activity, complement system, neuronal health, and excitatory and inhibitory synapse density and activity, in the DG of adult offspring exposed to mIA with poly I:C. Most of the observed changes were likely sex independent, including the alterations in C1q and C3 expression and levels; increase in dark neuron and dendrite density; and increase in mIPSC amplitude. However, a number of changes were sex specific, including the increase in CD68 and CD11b levels (both in general and following mIA) observed in female offspring, and the reorganization of inhibitory and excitatory synapses number and activity following mIA.

CD68 is a marker of phagolysosomal activity that is often used to assess the reactivity of microglia (Korzhevskii and Kirik, 2016). In our data, CD68 was exclusively expressed by IBA1+ cells, which likely represented microglia, but could also be infiltrating macrophages (González Ibanez et al., 2019). This is the first example of baseline sex differences in CD68 levels measured in the DG at adulthood; but there are previous reports of sex differences in which microglial CD68 is greater in females among hippocampus CA1 and forebrain regions earlier in development (Weinhard et al., 2018; Yanguas-Casás et al., 2018). Specifically, females had lower CD68 levels that increased with mIA, whereas males had stable levels that remained unchanged following mIA. This could indicate a ceiling, where the amount of CD68 is already higher in males, and thus is unable to manifest a sustained response to mIA at the time point analyzed in this study; or an earlier, transient change in phagolysosomal activity in males. Alternatively, we previously observed an increased density of dark microglia, which downregulate IBA1 and display high phagocytic activity, in males with mIA (Hui et al., 2018), it is possible that males could recruit different microglial subsets. This change in CD68 levels in females could be related to the mIA-induced reductions in excitatory synapses and mEPSC frequency.

In addition to mounting an increase in CD68 following exposure to mIA, females had greater CD11b levels, both basally and in response to mIA. This pattern was observed in the hippocampus of an Alzheimer’s disease model (Gallagher et al., 2013). CD11b is part of CR3, and phagocytosis can be triggered by iC3b’s (C3’s cleavage product) interaction with CR3 (Fu et al., 2012). In our work, we saw reductions in hippocampal expression of *C1q* and *C3* following mIA, across sexes, and also with protein levels of C1q, in neurons. These reductions could reflect a decrease in complement-mediated synaptic elimination (Stevens et al., 2007; Druart and Le Magueresse, 2019; Sellgren et al., 2019; Magdalon et al., 2020; Tenner, 2020). However, unlike the gene expression, C3 protein levels are increased with mIA across both sexes. These differences could highlight differences between DG and whole hippocampus; or could be protein driving gene regulation. C3 is located further downstream along the classical complement cascade, but is also activated by the alternative and lectin complement cascades (Druart and Le Magueresse, 2019). It is possible that increases in these multiple pathways drive synapse elimination differently across neuronal subtypes or regions. A parsimonious explanation may be that in males the mIA-induced reduction of neuronal C1q leads to reduced microglia-mediated synapse elimination (as inhibitory and excitatory synapses were more abundant in the PL with mIA), whereas in females there is an increase in C3, and greater still an increase in CD11b, that leads to elimination of excitatory synapses with mIA. Interestingly, C3 levels in our data were increased in GFAP+ astrocytes. Astrocytic C3 production may modulate synaptic density, as observed in models of Alzheimer’s disease (Lian et al., 2015, 2016; Luchena et al., 2018), amyotrophic lateral sclerosis (Heurich et al., 2011), perioperative neurocognitive disorder (Xiong et al., 2018) and diabetes (Zhao et al., 2018). We now show that there may be a similar phenomenon in the mIA model of schizophrenia.

As we previously observed altered microglial homeostasis and activation states (Hui et al., 2018), we investigated whether synaptic density changed after mIA. Imbalances in excitatory and inhibitory synaptic activities, notably in the hippocampus, are recognized as pathophysiological mechanisms in schizophrenia (Harrison et al., 2003; Coyle et al., 2012; Nakazawa et al., 2012; Tamminga et al., 2012; Li et al., 2015; Roberts et al., 2015; Matosin et al., 2016). In our data, for male offspring, we observed increased levels of mature inhibitory and excitatory synapses in the hippocampal DG, specifically the PL, following mIA. Furthermore, we saw an increase in only mIPSCs (amplitude and frequency) but not mEPSCs in DG GCs of male offspring. Therefore, it appears that despite potential increases in both mature inhibitory and excitatory synapses density in the PL, there is a net increase in functional inhibitory drive onto DG GCs in males. It is also possible that changes in excitatory synapses in the PL represent increased excitation of mossy cells or GABAergic interneurons that inhibit GCs (Scharfman, 2016). In addition, these data indicate that the perforant path, which is the major excitatory input to GCs (Witter, 2007; Treves et al., 2008), remains functionally unaltered in male offspring. Whereas in female animals, we observed no change in mature inhibitory synapses in the PL following mIA, but a potential reduction in mature glutamatergic synapses. There was also a concomitant increase in the amplitude of both mIPSCs and mEPSCs, and a decrease in mEPSC frequency. We cannot exclude a loss in functional excitatory synapses followed by a compensatory increase in the neurotransmitter content per quanta or in the density of the post-synaptic glutamate receptors (Pinheiro and Mulle, 2008) in female offspring. Future work is required to delineate specific changes with regards to pre- and post-synaptic site and specific inputs; as well as whether these effects are due to differences in microglial phagocytic activity. Additionally, as both excitatory and inhibitory currents on DG GCs are increased in females (perhaps compensatory evening out the output of these cells), but only inhibitory drive onto GCs is increased in males, this may contribute to sex differences in behavior observed following mIA (i.e., the prepulse inhibition deficits observed only in males) (Hui et al., 2018).

Our data reveals sex-dependent and -independent changes in the complement system and DG synapses following mIA. Despite some changes as a result of mIA, differences in other parameters may lead to sexual dimorphisms in structural and behavioral outcomes. For example, in males, changes in C1q but not CD68 could indicate less microglia-mediated synapse elimination, as there are perhaps increased synapse number in the PL and increased inhibitory input onto GCs. Contrastingly, in females, there were increases in CD68 and CD11b throughout the DG, potentially contributing to the potential reduction in excitatory synapses in the PL as well a decrease in GC mEPSC frequency, perhaps indicative of activity-dependent synaptic pruning. Through understanding the mechanisms underlying these sexual dimorphisms, we hope to provide new insights into novel therapies for schizophrenia or other diseases in which mIA is an environmental risk factor.

## Data Availability Statement

The raw data supporting the conclusions of this article will be made available by the authors, without undue reservation, to any qualified researcher.

## Ethics Statement

The animal study was reviewed and approved by the Université Laval’s Animal Ethics Committees, according to the Canadian Council on Animal Care’s guidelines.

## Author Contributions

CH, LS, LT, and M-ÈT designed the study. CH, XL, ÉG, LS, KB, and KS conducted the experiments. CH, HV, XL, ÉG, FM, LS, LT, and M-ÈT analyzed the data. CH, HV, LS, LT, and M-ÈT contributed to writing and editing the manuscript. CH, HV, and ÉG prepared the figures. All authors contributed to the article and approved the submitted version.

## Conflict of Interest

The authors declare that the research was conducted in the absence of any commercial or financial relationships that could be construed as a potential conflict of interest.

## Abbreviations

ACSF: artificial cerebrospinal fluid
C: complement
CD: cluster of differentiation
CR3: complement receptor 3
DAPI: 4′,6-diamidino-2-phenylindole
DG: dentate gyrus
E: embryonic
GABA: gamma aminobutyric acid
GC: granule cells
GFAP: glial fibrillary acidic protein
GL: granule cell layer
IBA: ionized calcium binding adaptor molecule
i.p.: intraperitoneal
MAP: microtubule-associated protein
mEPSC: miniature excitatory post-synaptic currents
mIA: maternal immune activation
mIPSC: miniature inhibitory post-synaptic currents
P: postnatal
PBS: phosphate buffered saline
PFA: paraformaldehyde
PL: polymorphic layer
poly I:C: polyinosinic:polycytidylic acid
ROI: region of interest
RT: room temperature
S: supplementary
SEM: standard error of the mean
TBS: tris buffered saline
TEM: transmission electron microscope
VGAT: vesicular GABA transporter
VGLUT: vesicular glutamate transporter
3-NT: 3-nitrotyrosine.
