# Supplementary material for: Sex Differences of Microglia and Synapses in the Hippocampal Dentate Gyrus of Adult Mouse Offspring Exposed to Maternal Immune Activation
Source: Front Cell Neurosci. 2020 Oct 15;14:558181. doi: 10.3389/fncel.2020.558181 (PMC7593822; doi:10.3389/fncel.2020.558181)
Supplement: Supplementary file 1 [file Data_Sheet_1.docx]

Supplementary Material

# Supplementary Materials and Methods

## Confocal Imaging for Synaptic Density and Staining Intensity Analysis

High resolution confocal images were acquired with a Carl Zeiss LSM-800 laser scanning confocal microscope in 3-4 mice per experimental group. After localization of the polymorphic layer (PL) inside dentate gyrus (DG) with exclusion of the CA3, two locations were imaged per brain section with a size of 101.4 x 101.4µm (1024x1024 pixels) using a Plan-Apochromat 63X/1.4 oil immersion objective. The two locations were taken at a similar position for each brain section. Each image was a series of 15+ *z*-stacks (step size ∆z = 0.33, averaged 2 times). The same settings were used during the imaging of each staining subset.

Synaptic puncta analysis was performed manually in ImageJ software using the Cell Counter plugin (National Institutes of Health). Two locations within the PL were analyzed per brain section and all locations were coded before the start of the analysis. Strict criteria were followed during the synaptic puncta counting, to ensure a consistent approach. As synaptic puncta counting is a subjective process, all analyses were performed blind to the experimental conditions.

For inhibitory puncta analysis, per location a region of interest (ROI) of 30x30µm was selected, which was determined with the 4’,6-diamidino-2-phenylindole (DAPI) channel only to avoid as much cell bodies as possible and to ensure an unbiased selection of ROIs. Afterwards, it was checked whether both the vesicular gamma aminobutyric acid (GABA) transporter (VGAT) and gephyrin staining was adequate within this area. Next, the 15 *z*-stacks with the most optimal staining were selected and a substack was created. In every ROI, first all VGAT puncta were counted throughout the 15 *z*-stacks and subsequently the same approach was followed for the gephyrin puncta. Finally, based on both channels and the individual VGAT and gephyrin counts, overlapping puncta were counted.

For excitatory puncta analysis, per location a ROI of 20x20µm was selected, which was also determined with the DAPI channel only to avoid as much cell bodies as possible and to ensure an unbiased selection of ROIs. Maximum intensity projections were created of the 4 z-stacks with the most optimal staining of vesicular glutamate transporter (VGLUT1) and Homer1. First, all Homer1 puncta were counted with the CellCounter plugin in ImageJ. Next, based on both channels and the individual Homer1 counting overlapping VGLUT1 and Homer1 puncta were counted with the 3D *z*-stack image next to it to verify actual overlap.

For the staining intensity analysis, the same mouse brain sections and locations were analyzed as in the synaptic puncta analysis. Both for the sections stained for inhibitory and excitatory synaptic proteins, per location 3 ROIs of 10x10µm were selected according to DAPI signals to ensure an unbiased selection of ROIs. Per ROI, 5 stacks were selected in which both VGAT and gephyrin puncta or both VGLUT1 and Homer puncta were clearly visible. After stacking these 5 images using ImageJ, the channels were split, and the average staining intensity was measured in the separate VGAT, gephyrin, VGLUT1, and Homer channels.

## Anatomical Analysis of Granule Cells

For post-hoc anatomical analysis, the neurons recorded during patch-clamp experiments were filled with biocytin (Sigma). Slices containing recorded cells were fixed overnight with paraformaldehyde (PFA, 4%) at 4°C, and then kept in phosphate buffer (0.1mM) containing sodium azide (0.03%). To reveal biocytin slices were permeabilized with triton X-100 (0.3%) and incubated for 1 hour in tris-buffered saline (TBS) containing normal donkey serum (20%). Then, slices were incubated with Alexa Fluor 488-conjugated streptavidin (1:1000, Jackson Immuno Research Laboratories, Inc.) for 24 hours at room temperature, rinsed and mounted on microscope slides with Fluoromount G fluorescence mounting medium (Southern Biotech). Confocal images of the neurons filled with biocytin were obtained using a Leica TCS SP5 imaging system coupled with a 488nm Argon laser. 3D Z-stacks (step-size: 1µm) were acquired using a 20× (NA, 0.8) oil-immersion objective.

GCs were examined quantitatively by measuring morphometric parameters such as soma area, number of branching points, total dendritic length, and total dendritic surface area. Sholl analysis was used to assess branch order distribution. All neurons were aligned to the node between the soma and the main dendrite, and concentric spheres, spaced 50 µm apart, were centered at this alignment node. The dendritic length and the number of branch points and nodes within each sphere was measured. All the above parameters were calculated by using Neuroexplorer in Neurolucida (MBF Bioscience).

# Supplementary Figures and Tables

## Supplementary Figures

### *Supplementary Figure 1. C1q puncta are not localized to IBA1+ microglia or GFAP+ astrocytes in the DG following mIA.*

**
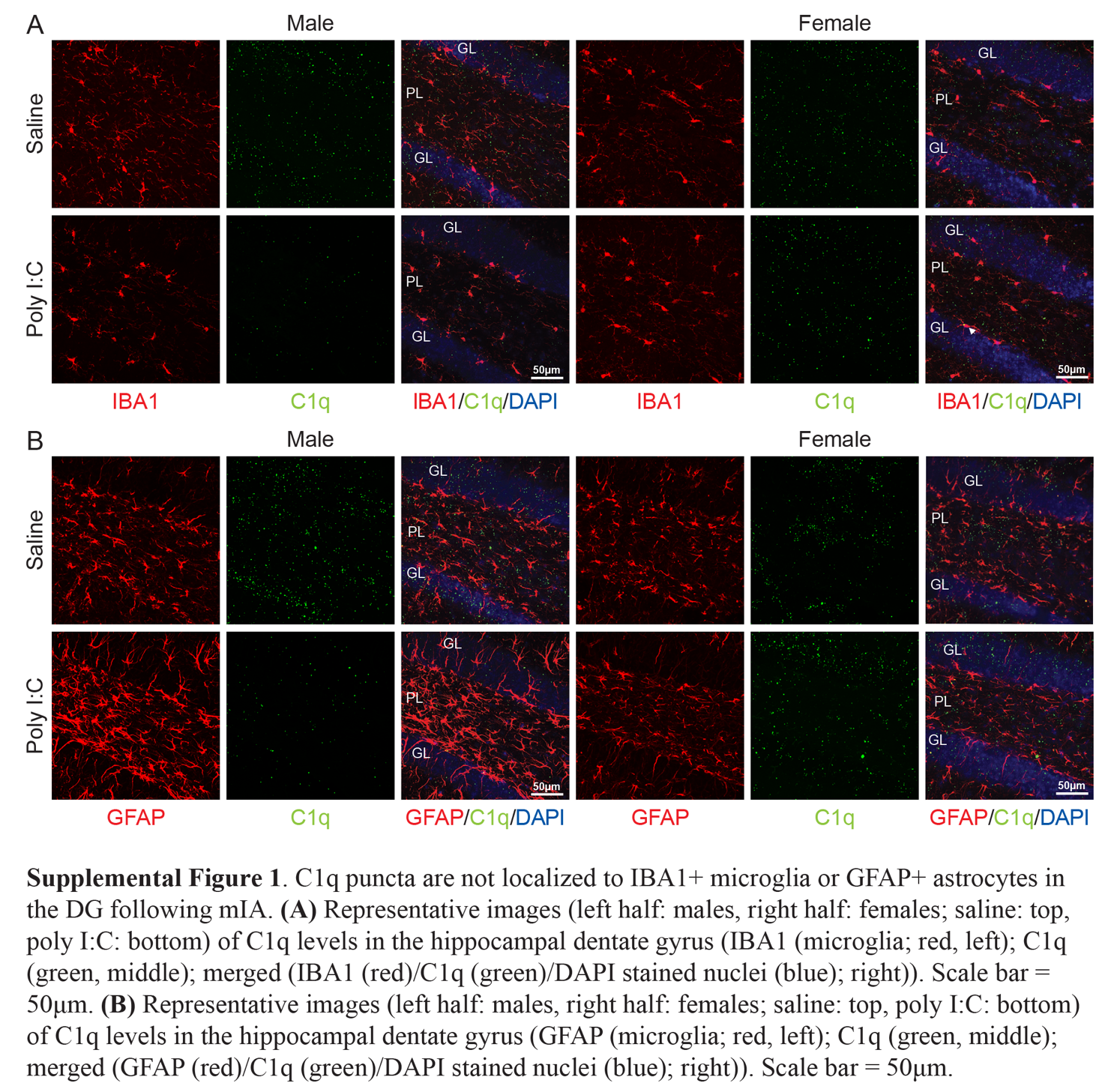
**

### *Supplementary Figure 2. Staining intensity of inhibitory and excitatory synapse markers are altered with mIA.*


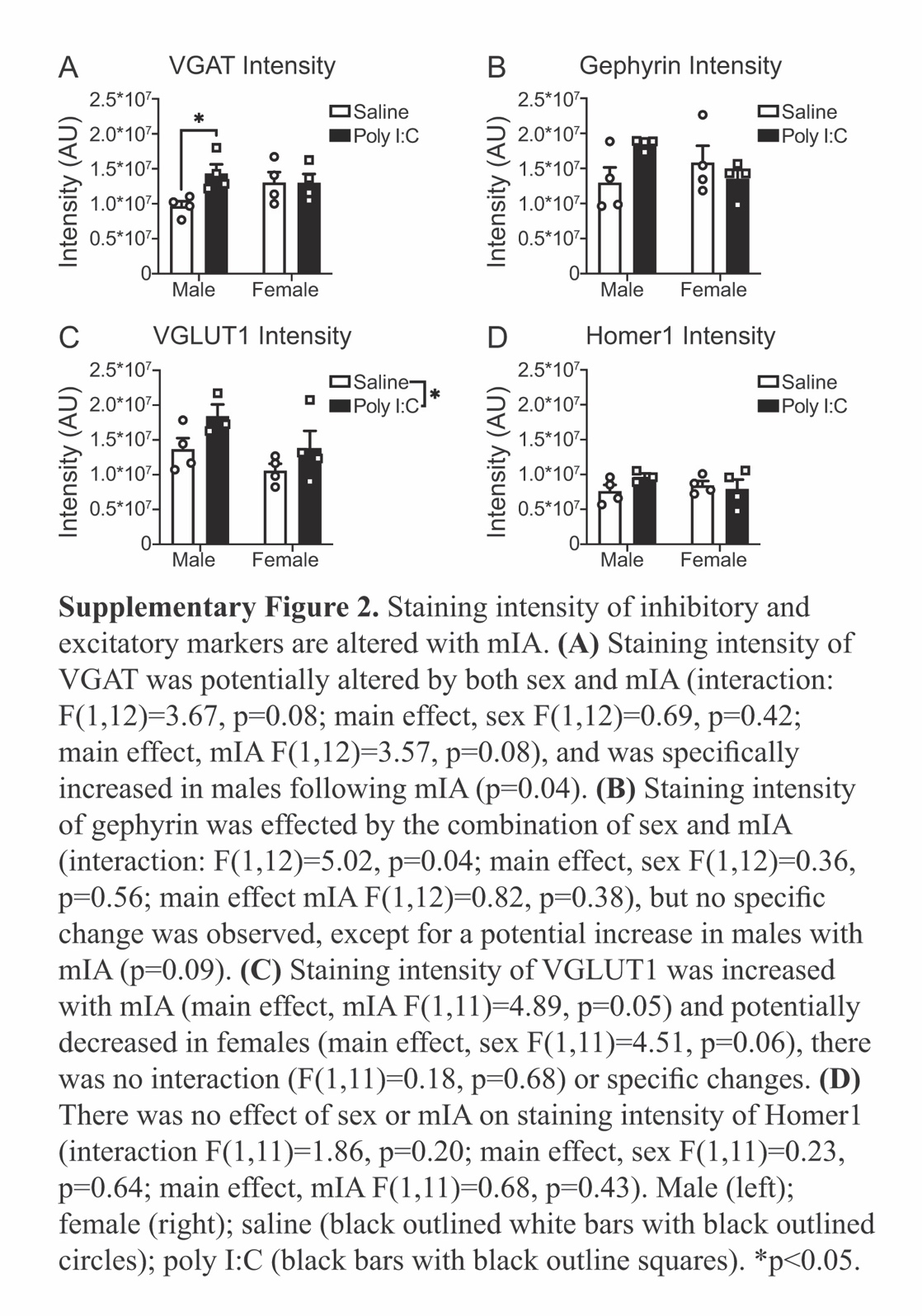


### *Supplementary Figure 3. Morphological properties of the DG GCs remain unaltered following mIA.*


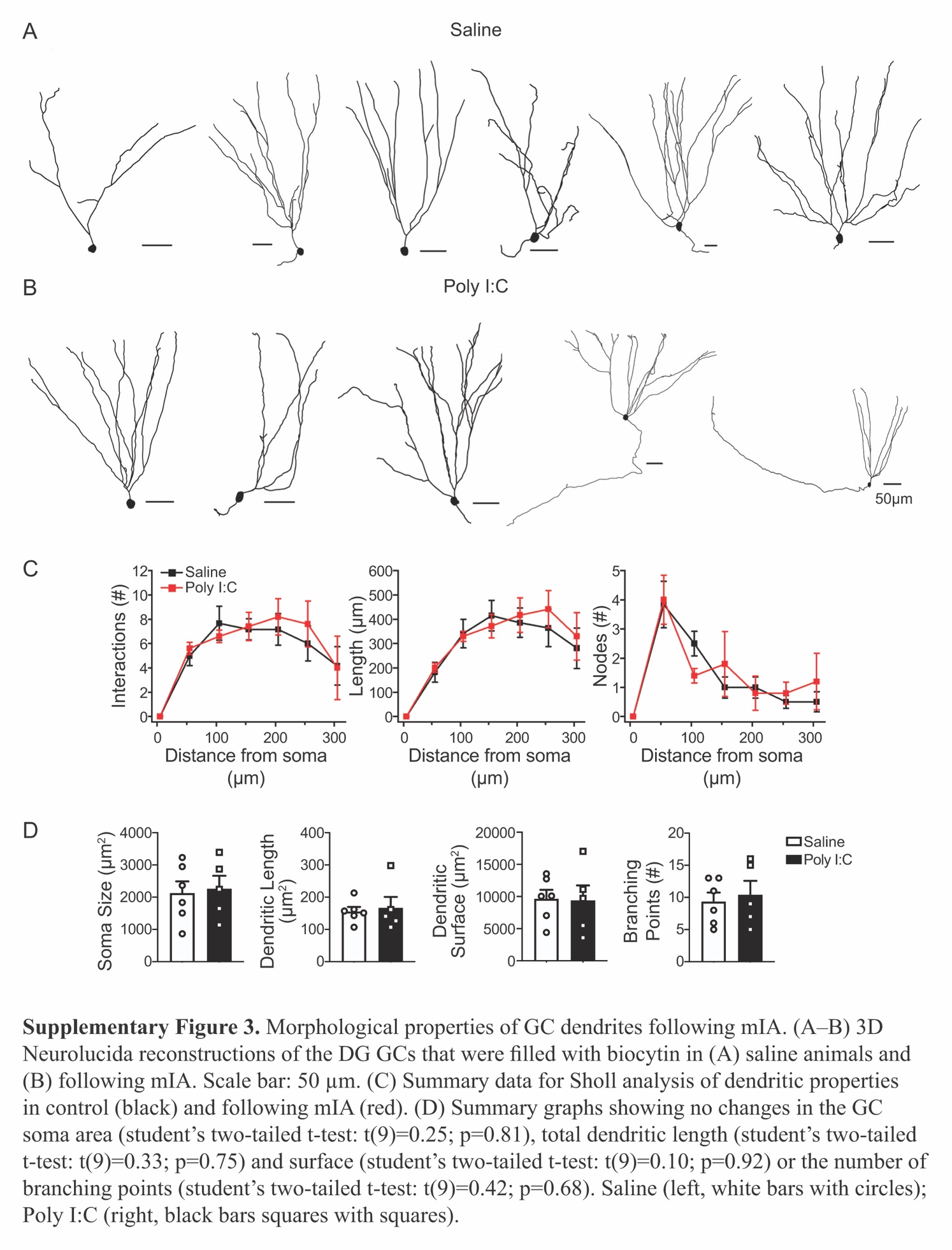


)

## Supplementary Tables

*1.2.1 Supplementary Table 1. Antibody Information for Immunofluorescence*

| Antibody | Order Information | Dilution | Blocking Buffer |
| --- | --- | --- | --- |
| Primary Antibodies | | | |
| rabbit polyclonal anti-IBA1 | Wako #019-19741 | 1:1000 | 10% donkey serum and 0.3% Triton X-100 in PBS |
| mouse monoclonal anti-IBA1 | Millipore MABN92 | 1:300 |  |
| rabbit polyclonal anti-GFAP | Abcam ab7260 | 1:2000 |  |
| rat monoclonal anti-CD11b | BioRad MCA111 | 1:1000 |  |
| rat monoclonal anti-CD68 | BioRad MCA1957 | 1:1500 |  |
| mouse monoclonal anti-C1q | Abcam ab71940 | 1:50 |  |
| rat monoclonal anti-C3 | Abcam ab11862 |  |  |
| chicken polyclonal anti-MAP2 | Abcam ab5392 | 1:2000 |  |
| rabbit polyclonal anti-3-NT | Millipore AB5411 | 1:400 |  |
| rabbit monoclonal anti-gephyrin | Synaptic Systems #147008 | 1:300 | 20% donkey serum and 0.3% Triton X-100 in PBS |
| mouse monoclonal anti-VGAT | Synaptic Systems #131011 | 1:500 |  |
| mouse monoclonal anti-VGLUT1 | Millipore MAB5502 | 1:5000 |  |
| rabbit polyclonal anti-Homer1 | Millipore ABN37 | 1:400 |  |
| Secondary Antibodies | | | |
| anti-rabbit AF488 | Jackson ImmunoResearch | 1:300 |  |
| anti-rat AF488 |  |  |  |
| anti-mouse AF488 |  |  |  |
| anti-rabbit AF568 |  |  |  |
| anti-rat AF568 |  |  |  |
| anti-mouse AF568 |  |  |  |
| anti-rabbit AF647 |  |  |  |
| anti-rat AF647 |  |  |  |
| anti-mouse AF647 |  |  |  |
| goat anti-rat biotin |  | 1:200 |  |

*1.2.2 Supplementary Table 2. Primer Sequences*

| Gene | Forward (5’-3’) | Reverse (3’-5’) |
| --- | --- | --- |
| Synaptic Development and Maintenance | | |
| *Stx1a* | cagggggagatgattgacagg | ctatccaaagatgcccccga |
| *Vamp2* | atctgagggtacagcccctt | cccagcatctctcctaccct |
| *Snap25* | caaggcgaacaactggaacg | gcttgttacagggacacacac |
| *Bdnf* | gacgacatcactggctgaca | caagtccgcgtccttatggt |
| *Fmr1* | agaaacctgaacccaaggct | tctctccaaacgcaactggt |
| *Shank2* | agccagaaagaaagctcccc | tgtccacgagttcctccaac |
| Phagocytosis and Synaptic Pruning | | |
| *Axl* | ttcaactgtgctacgtcccc | gggtccctctaggtaagcca |
| *MerTK* | ggacgttggtggatacgtgc | tctcttcccacttctcggca |
| *Gas6* | atgggtgcatgaggagttgg | tgttcgggtgtagttgagcc |
| *C1q* | aggactgaagggcgtgaaag | tggactctcctggttggtg |
| *C3* | ccccttaccccttcattcctt | agccgtaggacattgggagta |
| *C4a* | ggagcgcctacgaagactatg | gctccgacgaccctcaaata |
|  | | |
| Control | | |
| *Gapdh* | ggagaaacctgccaagtatga | ggtcctcagtgtagcccaag |

*1.2.3 Supplementary Table 3. Composition and Properties of the Solutions used for Acute Hippocampal Slice Preparation and for Electrophysiological Recordings*

| Sucrose-Based Perfusion Solution | *2mM KCl* |
| --- | --- |
|  | *1.25mM NaH_2_PO_4_* |
|  | *7mM MgSO_4_* |
|  | *26mM NaHCO_3_* |
|  | *10mM glucose* |
|  | *219mM sucrose* |
|  | *0.5mM CaCl_2_* |
|  | *pH 7.4* |
|  | *310 mOsm* |
| Heated Oxygenated Recovery Solution | *124mM NaCl* |
|  | *2.5mM KCl* |
|  | *1.25mM NaH_2_PO_4_* |
|  | *3mM MgSO_4_* |
|  | *26mM NaHCO_3_* |
|  | *10mM glucose* |
|  | *1mM CaCl_2_* |
|  | *pH 7.4* |
|  | *300 mOsm* |
|  | *35–37°C* |
| Oxygenated aCSF | *124mM NaCl* |
|  | *2.5mM KCl* |
|  | *1.25mM NaH2PO_4_* |
|  | *2mM MgSO_4_* |
|  | *26mM NaHCO_3_* |
|  | *10mM glucose* |
|  | *2mM CaCl_2_* |
|  | *pH 7.4* |
|  | *300 mOsm* |
|  | *95% O_2_/5% CO_2_* |
|  | *32±1°C* |
| Intracellular Solution | *130mM CsMeSO_4_* |
|  | *5mM CsCl* |
|  | *2mM MgCl_2_* |
|  | *10mM diNa-phosphocreatine* |
|  | *10mM HEPES* |
|  | *0.5mM EGTA* |
|  | *4mM ATP-TRIS* |
|  | *0.2mM GTP-TRIS* |
|  | *2mM QX-314* |
|  | *0.3% biocytin* |
|  | *pH 7.2–7.4* |
|  | *275–295 mOsm/L* |

*1.2.4 Supplementary Table 4. Gene Expression in the Hippocampus following mIA*

| Gene of Interest |  | Saline | Poly I:C | Statistics |
| --- | --- | --- | --- | --- |
| Synaptic Development and Maintenance | | | | |
| *Stx1a* | Male | 1.00±0.22, n=5 | 1.06±0.17, n=5 | interaction, F(1,16)=0.029, p=0.87;  main effect sex, F(1,16)=0.32, p=0.58;  main effect poly I:C, F(1,16)=0.27, p=0.61 |
|  | Female | 0.87±0.15, n=5 | 0.99±0.15, n=5 |  |
| *Vamp2* | Male | 1.00±0.12, n=5 | 1.03±0.14, n=5 | interaction, F(1,16)=0.03, p=0.86;  main effect sex, F(1,16)=0.03, p=0.86;  main effect poly I:C, F(1,16)=0.002, p=0.96 |
|  | Female | 1.05±0.16, n=5 | 1.03±0.17, n=5 |  |
| *Snap25* | Male | 1.00±0.27, n=5 | 0.88±0.22, n=5 | interaction, F(1,16)=0.35, p=0.56;  main effect of sex, F(1,16)=0.02, p=0.90;  main effect of poly I:C, F(1,16)=0.01, p=0.91 |
|  | Female | 0.89±0.22, n=5 | 1.06 0.26, n=5 |  |
| *Bdnf* | Male | 1.00±0.22, n=5 | 1.51±0.28, n=5 | interaction, F(1,14)=0.29, p=0.60;  main effect of sex, F(1,14)=0.91, p=0.36;  main effect of poly I:C, F(1,14)=2.74, p=0.12 |
|  | Female | 0.90±0.11, n=4 | 1.16±0.24, n=4 |  |
| *Fmr1* | Male | 1.00±0.15, n=5 | 0.98±0.15, n=5 | interaction, F(1,16)=0.07, p=0.79;  main effect of sex, F(1,16)=0.01, p=0.91; main effect of poly I:C, (F(1,16)=0.20, p=0.66) |
|  | Female | 1.06±0.08, n=5 | 0.95±0.16, n=5 |  |
| *Shank2* | Male | 1.00±0.16, n=5 | 0.97±0.21, n=5 | interaction, F(1,16)=0.08, p=0.79;  main effect of sex, F(1,16)=0.68, p=0.42; main effect of poly I:C, F(1,16)=0.02, p=0.88 |
|  | Female | 1.14±0.19, n=5 | 1.25±0.41, n=5 |  |
| Phagocytosis and Synaptic Pruning | | | | |
| *Axl* | Male | 1.00±0.05, n=5 | 0.96±0.06, n=5 | interaction, F(1,16)=0.003, p=0.96;  main effect of sex, F(1,16)=0.16, p=0.69;  main effect of poly I:C, F(1,16)=0.14, p=0.71 |
|  | Female | 1.03±0.13, n=5 | 1.00±0.12, n=5 |  |
| *MerTK* | Male | 1.04±0.15, n=5 | 0.94±0.15, n=5 | interaction, F(1,16)=0.006, p=0.94;  main effect of sex, F(1,16)=0.81, p=0.38;  main effect of poly I:C, F(1,16)=0.72, p=0.41 |
|  | Female | 1.17±0.14, n=5 | 1.05±0.07, n=5 |  |
| *Gas6* | Male | 1.00±0.19, n=5 | 0.98±0.15, n=5 | interaction, F(1,16)=0.11, p=0.75;  main effect of sex, F(1,16)=0.80, p=0.39;  main effect of poly I:C, F(1,16)=0.19, p=0.67 |
|  | Female | 1.21±0.21, n=5 | 1.08±0.12, n=5 |  |
| *C4a* | Male | 1.00±0.11, n=4 | 0.72±0.16, n=5 | interaction, F(1,15)=0.55, p=0.47;  main effect of sex, F(1,15)=0.03, p=0.87;  main effect of poly I:C, F(1,15)=0.22, p=0.65 |
|  | Female | 0.79±0.20, n=5 | 0.85±0.34, n=5 |  |

*1.2.5 Supplementary Table 5. Passive Membrane Properties of DG GCs following mIA*

| Parameter | Sex | Saline | Poly I:C | Statistics |
| --- | --- | --- | --- | --- |
| Resting membrane potential (mV) | Male | –73.4 ± 2.2, n=9 | –77.8 ± 2.0, n=5 | p=0.22 |
|  | Female | –75.7 ± 2.3, n=7 | –82.2 ± 1.1, n=11 | p=0.11 |
| Input resistance (MΩ) | Male | 161.9 ± 11.3, n=9 | 160.4 ± 31.3, n=5 | p=0.96 |
|  | Female | 190.2 ± 27.0, n=7 | 180.9 ± 11.5, n=11 | p=0.83 |
| Membrane capacitance (pF) | Male | 58.3 ± 6.3, n=9 | 64.6 ± 7.7, n=5 | p=0.55 |
|  | Female | 47.3 ± 9.3, n=7 | 51.8 ± 4.0, n=11 | p=0.84 |
